# *Daeshiho-tang* Attenuates Atherosclerosis by Regulating Cholesterol Metabolism and Inducing M2 Macrophage Polarization

**DOI:** 10.3390/life12020197

**Published:** 2022-01-28

**Authors:** Min-Young Song, Haneul Cho, Sora Lee, Kyung Hye Lee, Weon Kim

**Affiliations:** 1 Division of Cardiology, Department of Internal Medicine, Kyung Hee University Hospital, Kyung Hee University, Seoul 02447, Korea; kn60820@naver.com (M.-Y.S.); hn.cho1985@gmail.com (H.C.); trua1004@nate.com (S.L.); khl@cha.ac.kr (K.H.L.); 2 Department of Biotechnology, Cha University, Pocheon 11160, Korea

**Keywords:** atherosclerosis, *Daeshiho-tang*, M2 macrophage polarization, cholesterol metabolism, PPARγ signaling

## Abstract

Dyslipidemia, the commonest cause of cardiovascular disease, leads to lipid deposits on the arterial wall, thereby aggravating atherosclerosis. DSHT (*Daeshiho-tang*) has long been used as an anti-dyslipidemia agent in oriental medicine. However, the anti-atherosclerotic effects of DSHT have not been fully investigated. Therefore, this study was designed to evaluate whether DSHT could exert beneficial anti-atherosclerotic effects. We fed apolipoprotein E-deficient (ApoE-/-) mice on a high-fat diet and treated them with atorvastatin (AT) or DSHT, or the combination of DSHT and AT for 12 weeks. To determine the role of DSHT, atherosclerotic lesions in the aorta, aortic root, and aortic arch; lipids and apolipoprotein levels in serum; and macrophage polarization markers in aorta tissues were examined. We show here that the DSHT decreased the atherosclerotic plaque ratio in the aortic arch, aorta, and aortic root. DSHT also regulated lipid levels by decreasing the ApoB level and increasing the ApoA1 level. Moreover, DSHT effectively regulated cholesterol metabolism by increasing the levels of PPARγ, ABCA1 and ABCG1, and the LDL receptor genes. We further found that DSHT promoted polarization to the M2 phenotype by increasing the levels of M2 macrophage (ARG1, CD163, and PPARγ) markers. Our data suggested that DSHT enhances the anti-atherosclerotic effect by regulating cholesterol metabolism through the activation of the PPARγ signaling pathway and by promoting anti-inflammatory M2 macrophage polarization.

## 1. Introduction

Atherosclerosis is a chronic inflammatory disease characterized by lipid deposition, oxidative stress, foam cell formation, monocyte migration, and inflammatory response [1]. Because of these characteristics, many studies have focused on the absorption of lipids and inhibition of foam cell formation, which play pivotal roles in the early onset of atherosclerosis. In addition, cholesterol leakage is considered to play an important role in the formation of atherosclerotic plaques [2,3].

Current clinical guidelines widely recommend statins as the primary drugs for regulating lipid metabolism in atherosclerosis [4]. Many large-scale clinical trials have shown that statins substantially alleviate the progress of atherosclerosis, primarily because of their lipid-lowering effects [5]. Recently, combination therapy with drugs that can reduce the adverse effects while having properties similar to statins is becoming an increasingly used treatment strategy. Some combinational therapies, such as statin with ezetimibe, can reduce cholesterol absorption in the intestine by targeting the Niemann-Pick C1-like 1 protein. When ezetimibe is used in combination with statins, it can reduce low-density lipoprotein cholesterol (LDL-C) levels by an additional 25% [6]. Therefore, there is a critical need to find new drugs and treatment strategies for effective lipid regulation, with fewer adverse effects and improved safety.

Herbal medicines have been traditionally used and have received considerable attention as important agents for treating and preventing cardiovascular diseases such as atherosclerosis [7,8]. As a representative herbal medicine, *Daeshiho-tang* (DSHT) has been used in Korea for its preventive and treatment effects on diabetes [9], hepatotoxicity [10], hypertension, arterial constriction [11], and inflammatory reactions. DSHT is an herbal medicine that consists of six ingredients. Its ingredients have the following effects: saikosaponin from Bupleuri Radix (*Bupleurum falcatum* L.) improves triglyceride and LDL-C levels [12]; baicalin and wogonin from Scutellariae radix (*Scutellaria baicalensis* Georgi) increase HDL-C levels while decreasing total cholesterol/triglyceride levels [13]; sennoside A from Rhei Rhizoma (*Rheum palmatum* L.) decreases LDL-C, tumor necrosis factor-alpha (TNF-α), interleukin (IL)-6, and inflammatory cytokine levels [14,15]; Paeoniae Radix (*Paeonia lactiflora* Pall.) extract decreases LDL-C levels while increasing HDL-C levels [16]; and Ponciri Fructus Immaturus (*Poncirus trifoliata* (L.) Raf.) extract inhibits the activity of 3-hydroxy-3-methylglutaryl coenzyme A reductase (HMG-CoA) [17]. Moreover, ongoing studies on the mechanisms of intrahepatic lipid synthesis inhibition and adipocyte differentiation might provide evidence that DSHT regulates lipid metabolism. Furthermore, clinical studies on DSHT have revealed its important effect in reducing LDL-C levels without causing relevant changes in the liver and kidney functions in dyslipidemia [18].

However, how DSHT regulates lipid metabolism in atherosclerosis is still unclear. In addition, the potential use of the combination therapy of DSHT with statins is yet to be investigated. Therefore, this study aimed to investigate the effects of DSHT alone and in combination with AT, and the potential mechanisms underlying its effect by studying atherosclerotic plaque formation and changes in lipid metabolism and the balance of macrophage polarization in atherosclerosis-prone apolipoprotein E-deficient (ApoE-/-) mice fed with a high-fat diet (HFD).

## 2. Materials and Methods

### 2.1. Animals

Forty 8-week-old male ApoE gene knockout (ApoE-/-) B6.129P2-Apoetm1Unc/J mice (no. 002052) were purchased from The Jackson Laboratory (Bar Harbor, ME, USA). After 1 week of adaptive feeding, the mice were randomly assigned into four groups (n = 10 each). All mice were fed an HFD (D12079B; Central Lab. Animal Inc., Seoul, Korea) containing 21% fat and 1.5% cholesterol (Tables S1 and S2) [19] and exposed to the following interventions: control group (vehicle group), treated with normal saline; DSHT group, treated with 1000 mg/kg/day DSHT [20]; atorvastatin (AT) group, treated with 10 mg/kg/day AT [21]; DSHT and AT combination group (DSHT + AT group), treated with 1000 mg/kg/day DSHT and 10 mg/kg/day AT. AT was purchased from Pfizer (New York, NY, USA) and DSHT was purchased from Hanpoong Pharm. Co. Ltd. (Seoul, Korea). The composition of DSHT is shown in Table 1. Both agents were dissolved in normal saline and administered by gastric gavage (orally) at a fixed time. The experimental period was 12 weeks. All mice were housed in standard cages, with five mice in each cage in a room maintained at 22 ± 1 °C on a 12 h/12 h light/dark cycle with food and water available ad libitum. Body weight was recorded weekly. All procedures involving animals and their care were approved by the Institutional Animal Care Use Committee at Kyung Hee University Hospital (approval no. KHMC-IACUC18-020).

### 2.2. Quantification of Atherosclerotic Lesions

At the end of the experiment, all mice were sacrificed by injecting an overdose of sodium pentobarbital solution (40 mg/kg). The heart was fully perfused with phosphate-buffered saline (PBS) and carefully removed to analyze the aortic root lesions. Cryostat sections across the aortic root were prepared and embedded in an optimal cutting temperature compound. Serial sections (5 μm thick) were cut and collected until three valves appeared under the microscope. Five sections were placed onto each slide for a total of four slides. These slices were stained with Oil Red O (Sigma-Aldrich, St. Louis, MO, USA). A Zeiss confocal microscope was used to observe the pathological changes, and ImageJ software was used for quantifying the atherosclerotic lesions.

Between the aortic arch and the junction of the iliac arteries, the entire aorta was separated from the surrounding tissues under the dissecting microscope and fixed with 4% paraformaldehyde for 72 h. Thereafter, the aorta was longitudinally opened with ophthalmic scissors, horizontally tiled, and fixed using steel needles on the wax base of a black dish. The aorta was carefully washed three times with PBS to remove any floating impurities. Subsequently, the aorta was stained with Oil Red O for 1 h at room temperature, rinsed with PBS, and used to analyze the proportion of red lipid plaques in the entire aorta by using ImageJ software.

### 2.3. Measurement of Serum Cholesterol Parameters

After the sacrifice of mice, blood was collected from the heart, and the serum was separated and stored at −80 °C. The serum levels of ApoA1(ab238260) and ApoB(ab230932) were measured with an ELISA assay kit. All serum parameters were determined using the corresponding commercial kits from Abcam (Cambridge, UK) according to the manufacturer’s instructions; the intra- and inter-assay coefficients of variation (CV) of ApoA1 were 24% and 23.9% in the vehicle group, 6.1% and 6% in the AT group, 16.1% and 16% in the DSHT group, and 30% and 29.8% in the DSHT + AT group. The CV of ApoB were 18.9% and 16.4% in the vehicle group, 15.5% and 15.4% in the AT group, 10.2% and 10.1% in the DSHT group, and 17.8% and 17.7% in the DSHT + AT group. In addition, the CV of ApoB/ApoA1 ratio was 30.5% and 19% in the vehicle group, 6.3% and 5.9% in the AT group, 10.3% and 6.5% in the DSHT group, and 13.2 and 10.2% in the DSHT + AT group.

### 2.4. RNA Isolation and Quantitative qPCR Analysis

The total RNA from aorta tissues was extracted using TRIzol reagent (Invitrogen, Carlsbad, CA, USA), and reverse transcriptions were performed using the Accupower RocketScript Cycle RT Premix (dN12) kit according to the manufacturer’s instructions. Real-time polymerase chain reaction (PCR) was performed using the StepOnePlus Real-time PCR system (Applied Biosystems) with the Power SYBR Green Master Mix kit (Applied Biosystems, Foster, CA, USA). All samples were assayed in triplicate. Data were analyzed using the ΔΔCt method. Individual mRNA expression was normalized in relation to the expression of endogenous β-actin. PCR amplification consisted of 5 min of an initial denaturation step at 95 °C, followed by 46 cycles of PCR at 95 °C for 15 s and 60 °C for 30 s. The primers used for each gene are listed in Table 2.

### 2.5. Immunohistochemistry for Macrophages

For the immunohistochemical staining of aortic root sections, endogenous peroxidase activity was quenched with 3% hydrogen peroxide. Thereafter, the sections were incubated with primary antibodies at 4 °C overnight. Macrophages were detected using rabbit anti-mouse F4/80 antibody (Abcam, Cambridge, UK). IHC-Tek streptavidin-horseradish peroxidase solution (IHC World, Woodstock, MD, USA) and diaminobenzidine substrate (Vector Laboratories, Burlingame, CA, USA) were used to develop the brown reaction product. Slides were counterstained with hematoxylin. Pictures were obtained using a Zeiss confocal microscope. Images were analyzed using ImageJ software.

### 2.6. Statistical Analysis

Normality distribution was determined using the Shapiro–Wilk test. Two-tailed significant levels were fixed at the *p*-value of <0.05. Descriptive statistics were performed, the unpaired *t*-test and the one-way ANOVA were applied when normal distribution was found, and non-parametric tests (Mann–Whitney U) were applied when distribution was not normal (Shapiro–Wilk *p*-value < 0.05). Data are expressed as mean ± standard error of mean. All statistical analyses were performed using GraphPad Prism software (version 8.0; GraphPad Software Inc., La Jolla, CA, USA).

## 3. Results

### 3.1. General Observations

**Figure 1:**
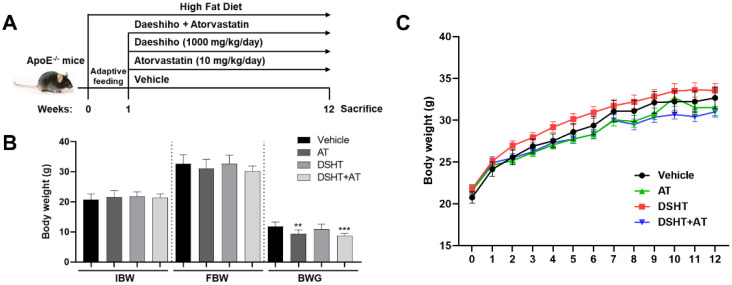
Effects of DSHT on growth of HFD-fed ApoE-/- mice. (**A**) Schematic diagram of the experimental schedule. The body weight of each group of mice was monitored once a week. (**B**) Growth curve of the mice. (**C**) Initial body weight (IBW), final body weight (FBW), and body weight gain (BWG) of the mice. Data are presented as mean ± standard error of mean of the samples from mice in the vehicle (n = 9), DSHT + AT (n = 9), AT (n = 10), and DSHT (n = 10) groups. ** *p* < 0.01; *** *p* < 0.001, compared with the vehicle group (unpaired *t*-test).

The effects and mechanisms of action of DSHT were studied in well-established atherosclerosis-prone ApoE-/- mice. All mice in this study were started HFD feeding a week prior to the administration of the vehicle, AT, DSHT, and DSHT + AT for 11 weeks (Figure 1A). The body weight (BW) of all mice gradually increased over time. The total BW measured every week increased at a similar rate in the DSHT group (3.25 ± 0.37%) and the vehicle group (3.64 ± 0.29%), whereas the AT group (2.96 ± 0.26%) and the DSHT + AT group (2.78 ± 0.15%) showed slightly lower growth rates (Figure 1C). The initial BWs and final BWs of the mice were not significantly different between the vehicle and experimental groups; however, significant differences between the vehicle group and the AT and DSHT + AT groups (*p* < 0.01) were observed in terms of BW gain (Figure 1B).

### 3.2. DSHT Reduces Atherosclerotic Plaques in the Aortic Arch

**Figure 2:**
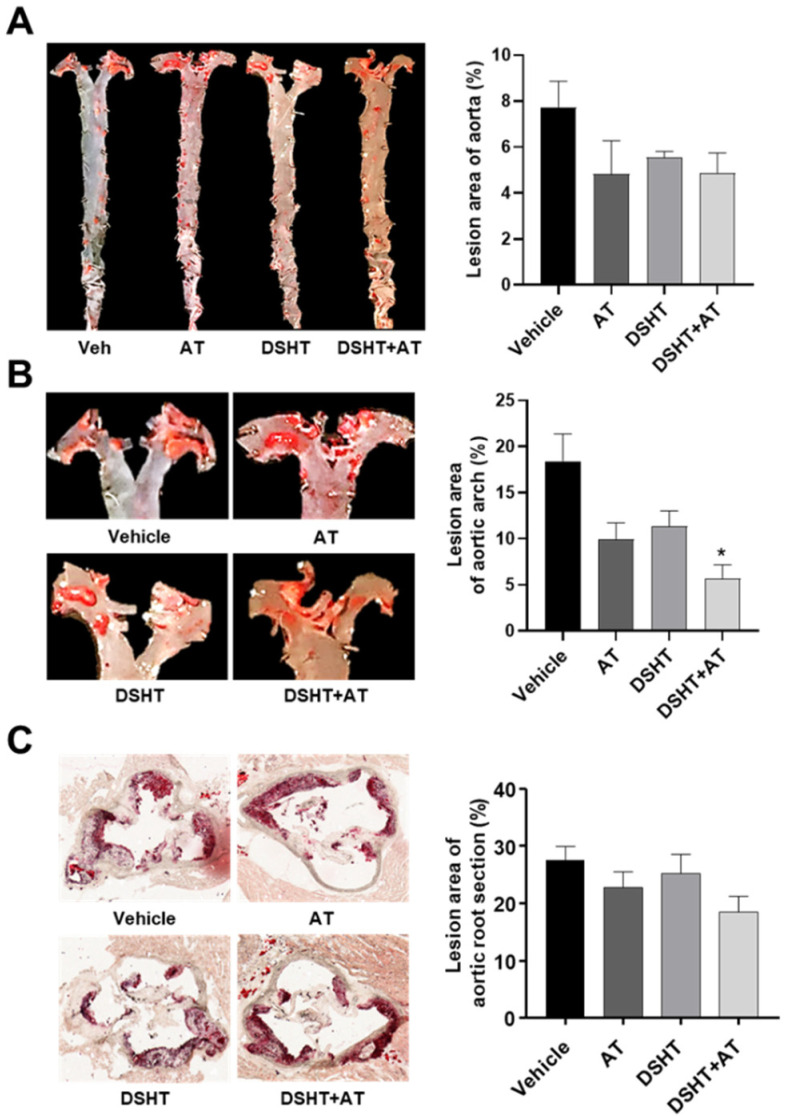
DSHT therapy attenuates atherosclerotic plaques in the aorta, aortic arch, and aortic root. (**A**) Between the aortic arch and the junction of the iliac arteries, en face aorta was separated from surrounding tissues and stained with Oil Red O in each group. (**B**) Plaque lesion analysis of the aortic arch (enlarged from the entire aorta). (**C**) To microscopically analyze the atherosclerotic lesions, sections from the aortic root were cut for paraffin embedding and stained with Oil Red O. The aorta en face and root sections were quantified using ImageJ software. Data are presented as mean ± standard error of mean of the samples from mice in the vehicle (n = 3), AT (n = 3), DSHT (n = 3), and DSHT + AT (n = 4) groups. * *p* < 0.05, compared with the vehicle group (Mann–Whitney U test).

ApoE-/- mice fed an HFD showed increased atherosclerotic plaques in the aorta. Therefore, reducing atherosclerotic plaques is an effective approach for treating atherosclerosis. Although atherosclerotic plaque lesions in the aorta showed a tendency to decrease in the AT, DSHT, and DSHT + AT groups compared with those in the vehicle group, no statistical significance was observed among all groups (Figure 2A). In the aortic arch, the area of atherosclerotic plaque lesion decreased in the AT and DSHT groups by 9% and 7%, respectively. Interestingly, the DSHT + AT group showed a further decrease of 13% in the plaque lesion area in the aortic arch compared with the vehicle group (*p* < 0.05) (Figure 2B). The area of plaque lesions in the aortic root in the AT and DSHT groups did not significantly differ from that in the vehicle group; however, the size of the lesions in the DSHT + AT group was additionally 10% lower than that in the AT and DSHT groups (Figure 2C).

### 3.3. DSHT Decreases Blood Lipid Level

**Figure 3:**
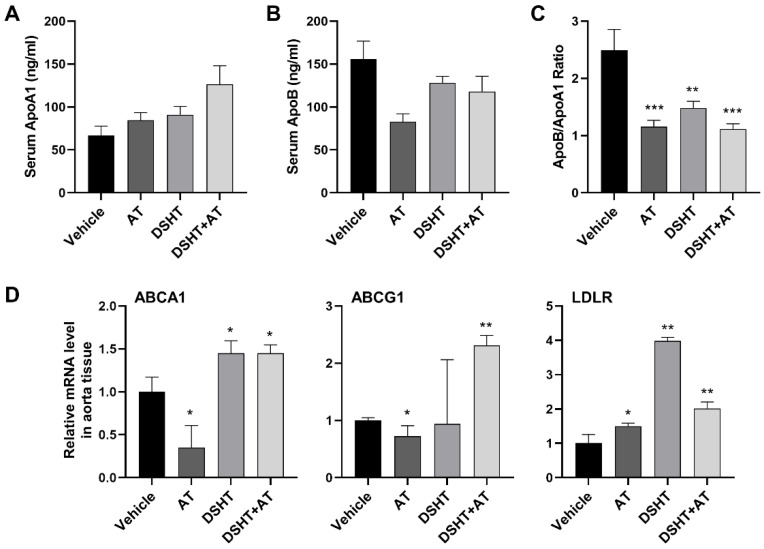
DSHT therapy promotes cholesterol metabolism in serum and enhances the expression of ABCA1, ABCG1, and LDLR in the aorta. Serum (**A**) ApoA1 and (**B**) ApoB levels, and (**C**) their ratio. (**D**) Relative mRNA expression levels of cholesterol metabolism-related ABCA1, ABCG1, and LDLR genes. Data are presented as mean ± standard error of mean of the samples from mice in the vehicle, AT, DSHT, and DSHT + AT groups (n = 5 for all). * *p* < 0.05; ** *p* < 0.01; *** *p* < 0.001, compared with the vehicle group (Mann–Whitney U test).

We investigated changes in lipid levels by using ApoA1, a known HDL major structural lipoprotein that serves as an effective lipid level marker and plays a central role in cholesterol metabolism, and ApoB, a major component of very-low-density lipoprotein, intermediate-density lipoprotein, and LDL. Serum ApoA1 level increased by 1.27- and 1.37-fold in the AT and DSHT groups, respectively, compared with that in the vehicle group. It further increased up to 1.89-fold in the DSHT + AT group (Figure 3A). In contrast to the serum ApoA1 level, the serum ApoB level decreased by 0.46 in the AT group and by 0.17- and 0.24-fold in the DSHT and DSHT + AT groups, respectively (Figure 3B). Further, the ratio of ApoB/A1, which is a strong predictor of cardiovascular diseases, significantly decreased in all experimental groups compared with the vehicle group (*p* < 0.001; Figure 3C).

In addition, we evaluated whether the ABCA1, ABCG1, and LDL receptor (LDLR) genes, which play pivotal roles in the regulation of cholesterol metabolism through cholesterol efflux and absorption, are regulated after DSHT and DSHT + AT treatments. ABCA1 and ABCG1 promote HDL-C efflux, and LDLR is involved in intracellular LDL uptake and degradation. The ABCA1 level increased by 1.45-fold in the DSHT group compared with the vehicle group (*p* < 0.05), and the ABCG1 level increased by 2.3-fold in the DSHT + AT group (*p* < 0.01). Furthermore, LDLR significantly increased by 3.98-fold in the DSHT alone group (*p* < 0.01). In the DSHT + AT group, LDLR was lower than that in the DSHT alone group but increased by 2.02-fold compared with the vehicle group (*p* < 0.01) (Figure 3D).

### 3.4. DSHT May Influence M2 Macrophage Polarization in Atherosclerosis

**Figure 4:**
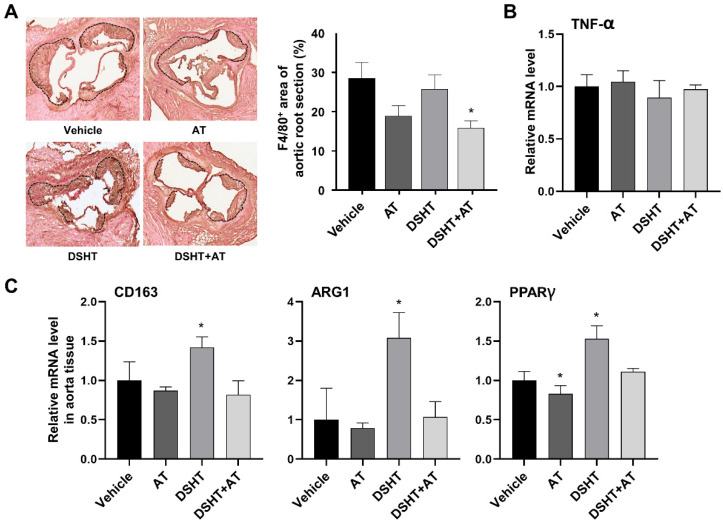
DSHT regulates M2 macrophage polarization in HFD-induced atherosclerosis ApoE-/- mouse model. (**A**) Representative histological analysis of cross-sections of the aortic root stained with F4/80 (macrophages). Right panels are the mean lesion area calculated from F4/80-positive aortic cross-sections. (**B**) Relative mRNA expression levels of the general inflammation and macrophage M1 phenotype (TNF-α) marker gene. (**C**) Relative mRNA expression levels of the macrophage M2 phenotype (CD163, ARG1, and PPAR) marker genes. Data are presented as mean ± standard error of mean of the samples from mice in the vehicle, AT, DSHT, and DSHT + AT groups (n = 5 for all). * *p* < 0.05, compared with the vehicle group (Mann–Whitney U test).

Macrophages play a pivotal role in the development and progression of atherosclerotic plaques, which are mediated by the pro-inflammatory M1 phenotype, and in the removal of atherosclerotic plaques, which is mediated by the anti-inflammatory M2 phenotype [22,23]. Hence, the polarization of M2 macrophages is an effective treatment approach for atherosclerosis. We examined the effect of DSHT and DSHT + AT on the phenotypic polarization of M1/M2 macrophages. Compared with the vehicle group, the AT and DSHT + AT groups showed a marked decrease in the polarization of macrophages, whereas the DSHT group showed slightly decreased polarization (Figure 4A). 

Thereafter, we examined the polarization status according to the M1/M2 phenotype. The TNF-α gene, a typical marker associated with inflammatory effects and the M1 phenotype, was maintained at a level similar to that of the vehicle group in all other groups (Figure 4B). The expression of the CD163, ARG1, and PPARγ genes, which are typical markers of the M2 phenotype, increased by 3.08-, 1.42-, and 1.53-fold in the DSHT group (*p* < 0.05) compared with that in the vehicle group, but decreased in the DSHT + AT group and was maintained at a level similar to that in the vehicle group (Figure 4C).

## 4. Discussion

Atherosclerosis is a progressive chronic inflammatory disease caused by maladaptive immune responses resulting from an imbalance in lipid metabolism and an accumulation of cholesterol-containing macrophages in the arterial wall. Recent evidence has suggested that DSHT has anti-hypertensive and anti-dyslipidemic properties that may contribute to the prevention of atherosclerosis [18]. In particular, the pharmacological effects of DSHT are derived from its main active ingredients [24], such as baicalin, saikosaponin A, sennoside B, and paeoniflorin, most of which have been clinically used because of their effects on lipid metabolism related to the risk of atherosclerosis [7]. Additionally, DSHT can affect other mechanisms associated with atherosclerosis, such as lipid accumulation in macrophages [25]. In this study, we used a well-established ApoE-/- mouse model to further investigate the role of DSHT in lipid metabolism, atherosclerotic plaque formation, and macrophage polarization, and obtained several new findings.

The overall effect of DSHT is metabolic regulation through lipid reduction [18]. In the current study, there was no statistically significant difference in body weight between the DSHT-treated mice and HFD-induced atherosclerosis models.

DSHT can be used from the early stages of atherosclerotic plaque formation and lipid deposition to reduce their progression. To clarify this hypothesis, we characterized atherosclerotic plaques in their important sites of occurrence: the aorta, aortic arch, and aortic root. We observed that treatment with DSHT relieved plaque formation more effectively in the aortic arch than in the aorta and aortic root. This is probably because of three reasons: (1) arterial relaxation through the regulation of nitric oxide and (2) atherosclerotic plaques generally appear earlier in the aortic arch than in the entire aorta [26] and (3) the plaque lesions assessed in our study were still in the early stages owing to our experimental design (mice were treated 1 week after they were fed an HFD) [27]. Our results are consistent with those of previous studies in which treatments with baicalin and saikosaponin, the major components of DSHT, were administered in early atherosclerosis stages and were absorbed by macrophages to relieve plaque lesions and lipid deposition through the reduction of oxidized LDL (ox-LDL), which is involved in foam cell formation [28,29].

Cholesterol metabolism plays a crucial role in the transformation of macrophages into foam cells. An abnormal increase in blood cholesterol and an imbalance in cholesterol metabolism are important factors contributing to the onset of atherosclerosis [30]. In the early stages of atherosclerosis, LDL oxidation by reactive oxygen species occurs along with DNA damage and changes in ox-LDL levels, which induce the progression of atherosclerotic plaques. In contrast, HDL plays a role in preventing the progression of atherosclerotic plaques by reversing cholesterol transport, thus transporting cholesterol from blood vessel macrophages to the liver [31,32]. We found that DSHT improved serum cholesterol metabolism by increasing the serum apoA-I level (HDL-C) while decreasing the serum apoB level (LDL-C) and calculated the ratio of ApoB/ApoA1 to establish that DSHT exhibits an anti-atherosclerotic effect.

The generally known major transporters involved in cholesterol efflux in macrophages are the ATP-binding cassette transporters ABCA1 and ABCG1 [33]. ABCA1 and ABCG1 are directly mediated by liver X receptor alpha (LXRα), a target gene for PPARγ [34,35]. Taken together, this suggests that cholesterol efflux occurs through the PPARγ-LXRα-ABCA1/G1 signaling process [36,37]. Another way of maintaining cholesterol metabolism is by inhibiting ox-LDL production in the early stages of atherosclerosis through removing LDL from the blood, thereby interfering with the differentiation of macrophages into foam cells. LDL enters macrophages through LDLR and subsequently undergoes the removal process [38,39]. As a limitation, the LDLR function is rapidly downregulated by cellular cholesterol accumulation [40]. In addition, LDLR levels increase depending on PPARγ activation [41]. Therefore, the production and activation of LDLR are crucial for attenuating atherosclerosis. 

Based on these diverse reports, we more specifically investigated the effect of DSHT on the expression of molecules involved in regulating cholesterol metabolism. As a result, we suggest that DSHT is involved in cholesterol efflux by promoting the expression of PPARγ, which acts at the top level of the PPARγ-LXRα-ABCA1/G1 pathway, then by regulating the transcription of ABCA1 in the DSHT group and ABCG1 in the DSHT + AT group. Moreover, DSHT may attenuate atherosclerosis by removing LDL from the blood through a marked increase in LDLR expression. These findings are consistent with the results of previous studies using baicalin and saikosaponin [28,29].

Macrophages are divided into the pro-inflammatory M1 and anti-inflammatory M2 phenotypes. In atherosclerosis, an excessive polarization of M1 macrophages increases the production of pro-inflammatory cytokines and promotes the destruction of atherosclerotic plaque fibrous tissue by continuously mediating the inflammatory response. In contrast, M2 macrophages increase the production of anti-inflammatory cytokines to promote the recovery of damaged tissues [42,43]. Furthermore, according to previous studies that examined macrophage phenotypes in ApoE-/- mice, those involved in atherosclerotic plaques had an M2 phenotype in the early stages but changed to an M1 phenotype as the lesion progressed [44]. Therefore, as strictly limiting M1 macrophages and increasing the polarization of M2 macrophages are important for attenuating atherosclerosis, we investigated the effect of DSHT on the polarization of M2 macrophages.

To examine the polarization of M2 macrophages by DSHT in detail, we assessed the gene expression of CD163 and ARG1, the representative markers of M2 macrophage polarization. As expected, their expression rapidly increased after treatment with DSHT. Interestingly, it has been reported that PPARγ, which increases the levels of ABCA1/G1 and LDLR (important molecules for cholesterol efflux and absorption, which are key regulatory processes in atherosclerosis), regulates the phenotypic polarization switch of M1/M2 macrophages and enhances the polarization of M2 macrophages [45,46]. It has also been shown that ApoA1 injection can significantly increase anti-inflammatory M2 macrophages and reduce inflammatory M1 macrophages in atherosclerotic plaques [47]. These results indicate that DSHT attenuates atherosclerosis by promoting M2 macrophage polarization and increasing the anti-inflammatory effect. To our knowledge, this study is the first to provide in vivo evidence supporting the anti-atherosclerotic activity of DSHT.

As an additional design point in this study, on the basis of the inhibitory effect of HMG CoA reductase activation by *Poncirus trifoliata* extract [48], a component of DSHT with the same mechanism of action as AT, we expected that the addition of DSHT to AT therapy would enhance the anti-atherogenic effect in a mouse model with HFD-induced atherosclerosis. We found that lipid deposition in the aorta and aortic arch, as well as the ratio of ApoB/ApoA1, decreased in the DSHT + AT group. However, the expression of ABCA1/G1 and LDLR, which affect the increase in HDL-C and the decrease in LDL-C, was lower in the DSHT + AT group than in the DSHT group. In addition, the combination treatment did not affect the polarization of M2 macrophages. Our results with the combination therapy have limitations for use in evaluating the synergistic effect of DSHT + AT in attenuating atherosclerosis, because the major components of DSHT can have adverse effects owing to drug–drug interactions. Thus, further studies are needed to explore potential signaling pathways by administering AT with single components of the herbs present in DSHT, in order to provide more compelling evidence for a new therapeutic approach.

## 5. Conclusions

Our study showed that DSHT regulates cholesterol metabolism by decreasing ApoB/ApoA1 levels and regulating the cholesterol metabolism-related genes ABCA1, ABCG1, and LDLR in an ApoE-/- mouse model of HFD-induced atherosclerosis. In addition, DSHT was shown to promote the polarization of M2 macrophages having anti-inflammatory effects by increasing the expression of CD163, ARG1, and PPARγ, which are M2 phenotypic marker genes. These results provide new insights into the effect of DSHT on cholesterol the metabolism and polarization of macrophages, which might offer a potential new approach for the treatment of atherosclerosis.

## Figures and Tables

**Table 1:** The composition of DSHT powder.

Botanic Name	Full Name	Relative Amount (g)	% of Total Weight
Bupleuri Radix	*Bupleurum falcatum* Linné	2.00	30
Pinelliae Tuber	*Pinellia ternata* Breitenbach	1.33	20
Scutellariae Radix	*Scutellaria baicalensis* Georgi	1.00	15
Paeoniae Radix	*Paeonia lactiflora* Pallas	1.00	15
Rhei Rhizoma	*Rheum palmatum* Linne	0.67	10
Ponciri Fructus	*Poncirus trifoliata* Rafinesque	0.67	10

**Table 2:** List of primers used for real-time PCR analysis.

Gene	Forward Primers	Reverse Primers
ABCA1	AAAACCGCAGACATCCTTCAG	CATACCGAAACTCGTTCACCC
ABCG1	GACACCGATGTGAACCCGTTTC	GCATGATGCTGAGGAAGGTCCT
LDLR	TGACTCAGACGAACAAGGCTG	ATCTAGGCAATCTCGGTCTCC
TNF-α	GCCTCTTCTCATTCCTGCTTG	CTGATGAGAGGGAGGCCATT
CD163	TCCACACGTCCAGAACAGTC	CCTTGGAAACAGAGACAGGC
ARG1	CTCCAAGCCAAAGTCCTTAGAG	AGGAGCTGTCATTAGGGACATC
PPARγ	TGGAATTAGATGACAGCGACTTGG	CTGGAGCAGCTTGGCAAACA

## Data Availability

Data are contained within the article.

## Supplementary Materials

The following supporting information can be downloaded at: https://www.mdpi.com/article/10.3390/life12020197/s1, Table S1: Formulation of diets; Table S2: Typical fatty acid composition of D12079B.

## Abbreviations

LDL-C	Low-density lipoprotein cholesterol
HDL-C	High-density lipoprotein cholesterol
DSHT	*Daeshiho-tang*
AT	Atorvastatin
Apo	Apolipoprotein
HFD	High-fat diet

## References

[1] Bäck M., Yurdagul A., Tabas I., Öörni K., Kovanen P.T. (2019). Inflammation and its resolution in atherosclerosis: Mediators and therapeutic opportunities. Nat. Rev. Cardiol..

[2] Moore K.J., Sheedy F.J., Fisher E.A. (2013). Macrophages in atherosclerosis: A dynamic balance. Nat. Rev. Immunol..

[3] Moore K.J., Tabas I. (2011). Macrophages in the pathogenesis of atherosclerosis. Cell.

[4] Stone N.J., Robinson J.G., Lichtenstein A.H., Merz C.N.B., Blum C.B., Eckel R.H., Goldberg A.C., Gordon D., Levy D., Lloyd-Jones D.M. (2014). 2013 ACC/AHA guideline on the treatment of blood cholesterol to reduce atherosclerotic cardiovascular risk in adults: A report of the American College of Cardiology/American Heart Association Task Force on Practice Guidelines. J. Am. Coll. Cardiol..

[5] Baigent C., Blackwell L., Emberson J., Holland L.E., Reith C., Bhala N., Peto R., Barnes E.H., Keech A., Cholesterol Treatment Trialists’ (CTT) Collaboration (2010). Efficacy and safety of more intensive lowering of LDL cholesterol: A meta-analysis of data from 170,000 participants in 26 randomised trials. Lancet.

[6] Cannon C.P., Blazing M.A., Giugliano R.P., McCagg A., White J.A., Theroux P., Darius H., Lewis B.S., Ophuis T.O., Jukema J.W. (2015). Ezetimibe Added to Statin Therapy after Acute Coronary Syndromes. N. Engl. J. Med..

[7] Kim J.Y., Shim S.H. (2019). Medicinal Herbs Effective Against Atherosclerosis: Classification According to Mechanism of Action. Biomol. Ther..

[8] Torres N., Guevara-Cruz M., Velazquez-Villegas L.A., Tovar A.R. (2015). Nutrition and Atherosclerosis. Arch. Med. Res..

[9] Jeong S.-M., Noh J.-W., Lee M.-S., Yang H.-G., Ahn Y.-M., Ahn S.-Y., Lee B.-C. (2020). A Retrospective Study on the Effect of Herbal Extracts Combined with Conventional Therapy on Blood Glucose in Type 2 Diabetes Mellitus. J. Intern. Korean Med..

[10] Kim C.-H., Kwon Y.-M., Lee Y.-T., Park S.-D. (2004). The preventive effect of Daeshiho-tang on liver damage induced by acetaminophen in the rats. Herb. Formula Sci..

[11] Yeo U.-H., Jo H.-J., Kim H.-H. (2005). Effects of Daesiho-tang extract on Hypertension and Arterial Contraction. J. Physiol. Pathol. Korean Med..

[12] Lee J.-C., Lee E. (2003). Effects of Siho (Bupleuri Radix) extracts on serum lipid composition and antioxidant system in rat fed high fat diet. Herb. Formula Sci..

[13] Lee H., Kang R., Hahn Y., Yang Y., Kim S.S., Cho S.H., Chung S.I., Yoon Y. (2009). Antiobesity effect of baicalin involves the modulations of proadipogenic and antiadipogenic regulators of the adipogenesis pathway. Phytother. Res..

[14] Zhang Y., Fan S., Hu N., Gu M., Chu C., Li Y., Lu X., Huang C. (2012). Rhein Reduces Fat Weight in db/db Mouse and Prevents Diet-Induced Obesity in C57Bl/6 Mouse through the Inhibition of PPARgamma Signaling. PPAR Res..

[15] Zhu X.L., Wang Y.J., Yang Y., Yang R.C., Zhu B., Zhang Y., Lin Y., Lu Y., Li X.F., O’Byrne K.T. (2012). Suppression of lipopolysaccharide-induced upregulation of toll-like receptor 4 by emodin in mouse proximal tubular epithelial cells. Mol. Med. Rep..

[16] Lee J.M., Choi S.W., Cho S.H., Lee S. (2003). Effect of Seeds Extract of Paeonia Lactiflora on Antioxidative System and Lipid Peroxidation of Liver in Rats Fed High-Cholesterol Dietentration Using a FIA Biosensor. Korean J. Nutr..

[17] Liu J.C., Chan P., Hsu F.L., Chen Y.J., Hsieh M.H., Lo M.Y., Lin J.Y. (2002). The in vitro inhibitory effects of crude extracts of traditional Chinese herbs on 3-hydroxy-3-methylglutaryl-coenzyme A reductase on Vero cells. Am. J. Chin. Med..

[18] Noh J.-W., Jeong S.-M., Kim D.-H., Yoo J.-H., Ahn Y.-M., Ahn S.-Y., Lee B.-C. (2019). A Retrospective Study on the Effect of Daeshiho-tang on the Lipid Profile in Patients with Uncontrolled Dyslipidemia by Statins. J. Intern. Korean Med..

[19] Li J., Lin S., Vanhoutte P.M., Woo C.W., Xu A. (2016). Akkermansia muciniphila protects against atherosclerosis by preventing metabolic endotoxemia-induced inflammation in Apoe^−/−^ mice. Circulation.

[20] Song M.-Y., Kim E.-K., Kong J.-C., Lee J.-H., Shin B.-C., Ryu D.-G., Kwon K.-B. (2008). Inhibitory effect of Daesiho-tang (Dachaihu-tang) extracts on high-fat diet-induced obesity. J. Korean Med. Rehabil..

[21] Roth L., Rombouts M., Schrijvers D.M., Martinet W., De Meyer G.R. (2016). Cholesterol-independent effects of atorvastatin prevent cardiovascular morbidity and mortality in a mouse model of atherosclerotic plaque rupture. Vasc. Pharmacol..

[22] Peled M., Fisher E.A. (2014). Dynamic Aspects of Macrophage Polarization during Atherosclerosis Progression and Regression. Front. Immunol..

[23] Randolph G.J. (2014). Mechanisms that regulate macrophage burden in atherosclerosis. Circ. Res..

[24] Kawashima T., Ogata M., Fujita N., Takahashi R. (2019). Daisaikoto Prevents Post-dieting Weight Regain by Reversing Dysbiosis and Reducing Serum Corticosterone in Mice. Front. Physiol..

[25] Yang S., Yuan H.Q., Hao Y.M., Ren Z., Qu S.L., Liu L.S., Wei D.H., Tang Z.H., Zhang J.F., Jiang Z.S. (2020). Macrophage polarization in atherosclerosis. Clin. Chim. Acta.

[26] Tomita H., Zhilicheva S., Kim S., Maeda N. (2010). Aortic arch curvature and atherosclerosis have overlapping quantitative trait loci in a cross between 129S6/SvEvTac and C57BL/6J apolipoprotein E-null mice. Circ. Res..

[27] Wang J., Xu P., Xie X., Li J., Zhang J., Wang J., Hong F., Li J., Zhang Y., Song Y. (2017). DBZ (Danshensu Bingpian Zhi), a Novel Natural Compound Derivative, Attenuates Atherosclerosis in Apolipoprotein E–Deficient Mice. J. Am. Heart Assoc..

[28] He X.W., Yu D., Li W.L., Zheng Z., Lv C.L., Li C., Liu P., Xu C.Q., Hu X.F., Jin X.P. (2016). Anti-atherosclerotic potential of baicalin mediated by promoting cholesterol efflux from macrophages via the PPARgamma-LXRalpha-ABCA1/ABCG1 pathway. Biomed. Pharmacother..

[29] He D., Wang H., Xu L., Wang X., Peng K., Wang L., Liu P., Qu P. (2016). Saikosaponin-a Attenuates Oxidized LDL Uptake and Prompts Cholesterol Efflux in THP-1 Cells. J. Cardiovasc. Pharmacol..

[30] Sukhorukov V.N., Khotina V.A., Chegodaev Y.S., Ivanova E., Sobenin I.A., Orekhov A.N. (2020). Lipid metabolism in macrophages: Focus on atherosclerosis. Biomedicines.

[31] Fotakis P., Kothari V., Thomas D.G., Westerterp M., Molusky M.M., Altin E., Abramowicz S., Wang N., He Y., Heinecke J.W. (2019). Anti-inflammatory effects of HDL (high-density lipoprotein) in macrophages predominate over proinflammatory effects in atherosclerotic plaques. Arterioscler. Thromb. Vasc. Biol..

[32] Barrett T.J., Distel E., Murphy A.J., Hu J., Garshick M.S., Ogando Y., Liu J., Vaisar T., Heinecke J.W., Berger J.S. (2019). Apolipoprotein AI) promotes atherosclerosis regression in diabetic mice by suppressing myelopoiesis and plaque inflammation. Circulation.

[33] Yvan-Charvet L., Wang N., Tall A.R. (2010). Role of HDL, ABCA1, and ABCG1 transporters in cholesterol efflux and immune responses. Arterioscler. Thromb. Vasc. Biol..

[34] Lee S.M., Moon J., Cho Y., Chung J.H., Shin M.J. (2013). Quercetin up-regulates expressions of peroxisome proliferator-activated receptor gamma, liver X receptor alpha, and ATP binding cassette transporter A1 genes and increases cholesterol efflux in human macrophage cell line. Nutr. Res..

[35] Larrede S., Quinn C.M., Jessup W., Frisdal E., Olivier M., Hsieh V., Kim M.J., Van Eck M., Couvert P., Carrie A. (2009). Stimulation of cholesterol efflux by LXR agonists in cholesterol-loaded human macrophages is ABCA1-dependent but ABCG1-independent. Arterioscler. Thromb. Vasc. Biol..

[36] Ogata M., Tsujita M., Hossain M.A., Akita N., Gonzalez F.J., Staels B., Suzuki S., Fukutomi T., Kimura G., Yokoyama S. (2009). On the mechanism for PPAR agonists to enhance ABCA1 gene expression. Atherosclerosis.

[37] Chawla A., Boisvert W.A., Lee C.H., Laffitte B.A., Barak Y., Joseph S.B., Liao D., Nagy L., Edwards P.A., Curtiss L.K. (2001). A PPAR gamma-LXR-ABCA1 pathway in macrophages is involved in cholesterol efflux and atherogenesis. Mol. Cell.

[38] Pirahanchi Y., Huecker M.R. (2018). Biochemistry, Ldl Cholesterol.

[39] Feingold K.R., Grunfeld C. (2015). Introduction to Lipids and Lipoproteins.

[40] Pennings M., Meurs I., Ye D., Out R., Hoekstra M., Van Berkel T.J., Van Eck M. (2006). Regulation of cholesterol homeostasis in macrophages and consequences for atherosclerotic lesion development. FEBS Lett..

[41] Duan Y., Chen Y., Hu W., Li X., Yang X., Zhou X., Yin Z., Kong D., Yao Z., Hajjar D.P. (2012). Peroxisome Proliferator-activated receptor gamma activation by ligands and dephosphorylation induces proprotein convertase subtilisin kexin type 9 and low density lipoprotein receptor expression. J. Biol. Chem..

[42] Duffield J.S. (2003). The inflammatory macrophage: A story of Jekyll and Hyde. Clin. Sci..

[43] Cardilo-Reis L., Gruber S., Schreier S.M., Drechsler M., Papac-Milicevic N., Weber C., Wagner O., Stangl H., Soehnlein O., Binder C.J. (2012). Interleukin-13 protects from atherosclerosis and modulates plaque composition by skewing the macrophage phenotype. EMBO Mol. Med..

[44] Khallou-Laschet J., Varthaman A., Fornasa G., Compain C., Gaston A.T., Clement M., Dussiot M., Levillain O., Graff-Dubois S., Nicoletti A. (2010). Macrophage plasticity in experimental atherosclerosis. PLoS ONE.

[45] Yao Q., Liu J., Zhang Z., Li F., Zhang C., Lai B., Xiao L., Wang N. (2018). Peroxisome proliferator–Activated receptor γ (PPARγ) induces the gene expression of integrin αVβ5 to promote macrophage M2 polarization. J. Biol. Chem..

[46] Wu H.M., Ni X.X., Xu Q.Y., Wang Q., Li X.Y., Hua J. (2020). Regulation of lipid-induced macrophage polarization through modulating peroxisome proliferator-activated receptor-gamma activity affects hepatic lipid metabolism via a Toll-like receptor 4/NF-κB signaling pathway. J. Gastroenterol. Hepatol..

[47] Sanson M., Distel E., Fisher E.A. (2013). HDL induces the expression of the M2 macrophage markers arginase 1 and Fizz-1 in a STAT6-dependent process. PLoS ONE.

[48] Huo X., Lu F., Qiao L., Li G., Zhang Y. (2018). A component formula of Chinese medicine for hypercholesterolemia based on virtual screening and biology network. Evid.-Based Complement. Altern. Med..

